# The relationship between TLR4/NF-κB/IL-1β signaling, cognitive impairment, and white-matter integrity in patients with stable chronic schizophrenia

**DOI:** 10.3389/fpsyt.2022.966657

**Published:** 2022-08-16

**Authors:** Hongna Li, Wenjin Chen, Mengzhuang Gou, Wei Li, Jinghui Tong, Yanfang Zhou, Ting Xie, Ting Yu, Wei Feng, Yanli Li, Song Chen, Baopeng Tian, Shuping Tan, Zhiren Wang, Shujuan Pan, Na Li, Xingguang Luo, Ping Zhang, Junchao Huang, Li Tian, Chiang-Shan R. Li, Yunlong Tan

**Affiliations:** ^1^Peking University HuiLongGuan Clinical Medical School, Beijing Huilongguan Hospital, Beijing, China; ^2^Department of Psychiatry, Yale University School of Medicine, New Haven, CT, United States; ^3^Department of Physiology, Faculty of Medicine, Institute of Biomedicine and Translational Medicine, University of Tartu, Tartu, Estonia

**Keywords:** schizophrenia, Toll-like receptor 4, inflammation, cognition, white matter

## Abstract

**Objective:**

Previous studies have implicated intricate interactions between innate immunity and the brain in schizophrenia. Monocytic Toll-like receptor (TLR) 4 signaling, a crucial “sensor” of innate immunity, was reported to be over-activated in link with cognitive impairment in schizophrenia. As TLR4 is predominantly expressed on gliocytes prior to expression in neurons, we hypothesized that higher TLR4 levels may contribute to cognitive deterioration by affecting white matter microstructure.

**Methods:**

Forty-four patients with stable chronic schizophrenia (SCS) and 59 healthy controls (HCs) were recruited in this study. The monocytic function was detected with lipopolysaccharide (LPS) stimulation to simulate bacterial infection. Basal and LPS- stimulated levels of TLR4, nuclear factor-kappa B (NF-κB), and interleukin (IL)-1β were quantified with flow cytometry. Cognitive function was assessed by the MATRICS Consensus Cognitive Battery (MCCB) and psychopathological symptoms were evaluated by the Positive and Negative Syndrome Scale (PANSS). We employed diffusion tensor imaging with a 3-T scanner and evaluated white-matter integrity with fractional anisotropy (FA). Subcortical volume and cortical thickness were also assessed.

**Results:**

The TLR4/NF-κB/IL-1β signaling pathway was activated in patients with SCS, but responded sluggishly to LPS stimulation when compared with HCs. Furthermore, monocytic TLR4 expressions were inversely correlated with cognitive function and white matter FA, but not with cortical thickness or subcortical gray matter volume in schizophrenia.

**Conclusion:**

Our findings support altered TLR4 signaling pathway activity in association with deficits in cognition and white matter integrity in schizophrenia.

## Introduction

The pathophysiology of schizophrenia remains an active focus of investigation. The intricate interactions between innate immunity and the brain may conduce to the development of schizophrenia (1). Higher levels of pro-inflammatory cytokines in pueritia related to increased risk of psychosis in adulthood (2). Clearance of some antibodies against synaptic surface receptor by plasmapheresis appeared significantly improved clinical symptoms in patients with schizophrenia (3). Notably, the Toll-like receptor (TLR) 4 signaling pathway is involved in innate immunity dysfunction and may elevate the vulnerability to schizophrenia (4, 5).

The Toll-like receptor 4, a pattern recognition receptor, is detected in conserved molecules or extracellular structures, termed pathogen-associated molecular patterns (PAMPs) and endogenous damage-associated molecular patterns (DAMPS). TLR4 plays a crucial role in immunosurveillance during innate immunity responses, constituting the first-line defense against pathogens (6). The TLR4 signaling pathway can be specifically triggered by lipopolysaccharide (LPS) (7), an endotoxin from the Gram-negative bacteria, resulting in myeloid differentiation primary response protein 88 (MyD88) dependent or MyD88-independent pathway activity that releases downstream pro-inflammatory cytokines, such as interleukin (IL)-1β and tumor necrosis factor (TNF)-α, to eliminate infection (8). However, the current research on TLR4 actions predominantly targets peripheral immune-related cells, including macrophages, monocytes, endothelial cells, and granulocyte cells (9). The expression of TLR4 was also observed in mammalian glial cells such as oligodendrocytes, astrocytes, and especial microglia (10), which is critical to neuroplasticity including synaptic remodeling, myelinated axons formation and neurogenesis, affecting learning and memory, as well as other aspect of cognition (10–13). Higher TLR4/NF-κB signaling in the brain has been related to the generation of subtle neuroinflammation and neural degeneration, contributing to the pathogenesis of schizophrenia (14, 15).

Previous studies have suggested dampened monocytic TLR4 activation during LPS stimulation that is manifested by less increases in the levels of proinflammatory cytokines, such as IL-1β and IL-6, leading to a weakened capability to eliminate pathogens and potentially sustained infections in schizophrenia (16–18). Currently, the correlations between TLR4 levels and cognitive performance in schizophrenia have not been fully elucidated, with complex and less than consistent results (5, 6, 17, 19). Some demonstrated that TLR4 may play a pathogenic role in cognitive dysfunction (17), whereas others showed negative findings (16).

Imaging studies with diffusion tensor imaging (DTI) showed that schizophrenia, especially treatment-resistant schizophrenia, is associated with region-specific patterns of white-matter deficits (20–22), although the potential mechanisms are as yet unclear. A postmortem study revealed microglia activation specifically in the frontal and temporal white-matter regions (23). Therefore, we speculated that there is a potentially etiological relationship between TLR4 signaling activity dysfunction and white-matter deficit in patients with schizophrenia.

In the current study we tested the following hypothesis: (a) The TLR4/NF-κB/IL-1β pathway is over-activated and its response is blunted to LPS stimulation in patients with schizophrenia; and (b) TLR4 pathway activation is prominently correlated with white-matter deficits and cognitive impairment in schizophrenia. In addition, regional cortical thickness and subcortical gray matter structures were extracted to check whether the activation of the TLR4 pathway was located in a specific white matter microstructure or extended throughout the whole brain.

## Methods

### Subjects

Forty-four patients with stable chronic schizophrenia (SCS) and 59 healthy controls (HCs) were recruited for the study. All patients visited Beijing Huilongguan Hospital as inpatients or outpatients between 2017 and 2018, and HCs were recruited from the community through advertising. Patients with SCS were of 18–65 years in age and Han nationality; meeting the *Diagnostic and Statistical Manual of Mental Disorders*, Fourth Edition, criteria for schizophrenia; and clinically stable on medication, defined as taking a steady dose of antipsychotics for at least 6 months.

Common exclusion criteria for patients and controls included: any severe, acute, or uncontrollable physical or neurological disorder; any chronic infection, autoimmune diseases, or use of immunosuppressants medication in the last 6 months; a history of alcohol or substance abuse or dependence; lactating or pregnant females; intellectual disability or inability to understand the experimental content.

This research followed the Helsinki Declaration and was approved by the Ethics Committee of Beijing Huilongguan Hospital. All participants provided written informed consent prior to study.

### Symptom and cognitive evaluations

Positive and Negative Syndrome Scale (PANSS) was conducted by two experienced psychiatrists to evaluate psychopathology with a coefficient of interclass correlation >0.8. The MATRICS Consensus Cognitive Battery (MCCB) was used for cognitive function evaluation in patients with schizophrenia (24) and a Chinese version of MCCB was developed due to differences in translation and culture. The Chinese-normalized T-scores for MCCB have been adjusted by gender, age, education and region (25). The retest reliability of the Chinese-version MCCB was (0.60–0.85:0.69–0.85); the inter-rater reliability was 0.97; the ceiling effect was 0.85% and floor effect was 1.72%, suggesting that the Chinese version of MCCB is more sensitive and applicable (26). The MCCB contains 10 tests covering seven different cognitive dimensions and Chinese-normalized T-scores were used for statistical analysis.

### Quantification of the TLR4, NF-κB, and IL-1β levels in CD14+ monocytes

The detailed experimental procedures can be found in our previous studies (16, 17) and main reagent information was shown in Supplementary Table 2. Serum samples (5 ml) from all participants between 6 and 7 am were collected. Whole blood samples (100 μl) were transferred into polystyrene FACS tubes and stained with 10 μl FITC-labeled mouse anti-human CD14. To quantify the TLR4, NF-κB and IL-1β levels, counterpart antibodies were added to each tube while corresponding isotype antibodies were added to the corresponding samples as negative controls to ensure staining specificity. Notably, additional Monensin, a Golgistop Protein transport Inhibitor, was required for IL-1β to enhance intracellular concentration and facilitate detection by flow cytometry.

Notably, NF-κB is a heterodimer composed of p50 and p65 subunits and activation of NF-κB requires phosphorylation in the transactivation domain of p65. When activated, NF-κB translocates to the nucleus and binds to specific DNA sequences to regulate the release of inflammatory cytokines (27, 28). Because flow cytometry detects the phosphorylation level of NF-κB p65 subunit with Phosflow™ PE Mouse Anti- NF-κB p65, it can indirectly reflect the activated levels of NF-κB in our study.

For LPS stimulation group, another serum samples (100 μl) were stimulated with LPS (100 ng/ml). Besides, the stimulation time of TLR4 and IL-1β for 5h and NF-κB for 5 min at 37°C were optimal (17). The following operation steps were in accordance with the previous instructions. Homotypic controls and corresponding serum samples without LPS stimulation were taken as the control group. We obtained a total of 2,500 events for each sample and used FlowJo V10 software for data analysis.

### Imaging and data processing

Imaging data were obtained using 3-T Prisma MRI scanner (Siemens, Germany), equipped with a 64-channel radio frequency head coil from Beijing Huilongguan Hospital Medical Imaging Center. DTI data were acquired using a spin-echo, echo-planar imaging sequence with a spatial resolution of 1.7×1.7×1.7 mm^3^, repetition time (TR) = 8,000 ms, echo time (TE) = 87 ms, field of view (FOV) = 224×224 mm^2^, 98 isotopically distributed diffusion-weighted directions and axial slice orientation with 82 slices and no gaps, 2 diffusion weighting values [b = 0 and 1,000 s/mm^2^], and 5 b = 0 images). Head movements were restricted with padding for participants and the ENIGMA-DTI analysis pipeline was conducted for processing DTI data. All data for our study passed ENIGMA-DTI quality guarantee and control. Regional white matter fractional anisotropy (FA) of 21 brain regions were generated each averaged across cerebral hemispheres based on the guidelines of ENIGMA-DTI atlas (29).

Structural T1-weighted imaging data were collected with a sagittal 3D-magnetization-prepared rapid acquisition gradient echo sequence (MP-RAGE), to assess subcortical volumes and cortical thickness. The scanning parameters were: TE = 2.98 ms, FOV= 256 × 224 mm^2^, TR = 2,530 ms, inversion time (TI) = 1,100 ms, matrix size = 256 ×224, flip angle = 7 and thickness/gap = 1/0 mm. Voluminal operation was conducted with FreeSurfer, version 5.3. We derived a volume score for nine subcortical structures, including accumbens, putamen, thalamus, caudate, amygdala, hippocampus, lateral ventricle, pallidum, lateral ventricle choroid plexus in addition to total intracranial volume (ICV). Seventy cortical thickness regions were extracted for analysis complying with the Desikan–Killiany atlas (30).

### Statistical methods

Student's *t-*test or chi-squared test was performed to compare demographics and TLR4, NF-κB and IL-1β levels between patients and controls. As the levels of IL-1β did not exhibit normal distribution, and were transformed by log10. Analysis of covariance (ANCOVA) was conducted to compare the MCCB scores, white-matter FA, subcortical volumes, and cortical thickness between patients and controls, with education level and ICV as covariates, respectively. Paired *t-*test was performed to evaluate the differences before and after LPS stimulation and ANCOVA was used to compare inflammatory markers levels after LPS stimulation between patients and controls, with respective unstimulated levels as covariates. Partial correlations were conducted between TLR4 levels and regional FA, subcortical volumes as well as cortical thickness in the patients, controlling for age, sex distribution and ICV. The relationship between TLR4 levels and PANSS scores as well as MCCB scores were assessed by partial correlation analysis, adjusting for age, sex, chlorpromazine (CPZ) equivalents and education level, respectively. To further explore the causal relationship among the TLR4 signaling pathway, cognitive performance and whole-brain average FA, we attempted to run a mediation effect analysis by SPSS's PROCESS macro. In the mediation model, we took TLR4 level as an independent variable; and processing speed of MCCB as a dependent variable and average FA as a mediator, respectively. Age, gender, education level and ICV were controlled as covariates. We chose Model 4 in the PROCESS and used the bootstrap method to test the significance of the mediating effect. The 95% confidence interval (CI) was displayed for direct, indirect and total effects with 5,000 bootstrap samples. If the 95% CI of the indirect effect did not contain 0, it meant that the mediating effect was significant (*p* < 0.05). All findings were considered statistically significant with *p* < 0.05 (2-tailed) with Bonferroni correction for multiple comparisons.

## Results

### Demographic and clinical characteristics

There were no differences in age, sex ratio, or education level between the schizophrenia patients and healthy controls. The illness duration was (22.91 ± 12.19) months, age of onset was(24.11±5.92)years and CPZ equivalents were (395.50 ± 219.06) mg/day. The PANSS scores were as follows: total (50.64 ± 10.67); positive (11.27 ± 4.05); negative (15.07 ± 5.65); and general (24.32 ± 3.64), indicating stable psychopathology in patients with SCS. An overall cognitive impairment following the seven domains of MCCB was found in SCS (*p* < 0.05/7 = 0.007, Table 1). Further analysis revealed no significant relationship between TLR4 levels and age of onset, illness duration, or CPZ medication in SCS.

**Table 1:** Demographics of participants and clinical characteristics of patients.

**Characteristics**	**SCS (*n* = 44)**	**HCs (*n* = 59)**	***x* ^2^ */t/F***	***p***
Gender, M/F	29/15	28/31	3.5	0.062
Age (yrs)	47.02 ± 10.87	43.54 ± 11.38	−1.56	0.121
Education (yrs)	12.34 ± 3.29	12.24 ± 2.41	−0.19	0.854
Illness duration (ms)	22.91 ± 12.19	NA	NA	NA
Age of onset (yrs)	24.11 ± 5.92	NA	NA	NA
CPZ equivalents (mg/day)	395.50 ± 219.06	NA	NA	NA
PANSS-score				
PANSS-total score	50.64 ± 10.67	NA	NA	NA
PANSS-positive	11.27 ± 4.05	NA	NA	NA
PANSS-negative	15.07 ± 5.65	NA	NA	NA
PANSS-general	24.32 ± 3.64	NA	NA	NA
MCCB score				
MCCB total score	45.33 ± 10.53	57.56 ± 8.69	6.12	2.00 × 10^−8^^***^
Speed of processing	42.36 ± 9.18	54.82 ± 8.66	6.89	1.00 × 10^−8^^***^
Attention/vigilance	45.69 ± 9.49	57.09 ± 9.15	5.96	4.00 × 10^−8^^***^
Working memory	46.33 ± 10.08	58.07 ± 8.22	6.23	1.00 × 10^−8^^***^
Verbal learning	50.21 ± 11.53	58.11 ± 8.50	3.92	1.63 × 10^−4^^***^
Visual learning	45.05 ± 11.68	52.39 ± 8.63	3.36	0.003^***^
Social cognition	46.33 ± 10.08	51.94 ± 9.77	2.72	0.008^**^
Reason and problem solving	42.85 ± 9.36	54.81 ± 8.66	6.89	1.00 × 10^−8^^***^

SCS, Stable Chronic Schizophrenia; HCs, Healthy Controls; NA, Not Applicable; MCCB, MATRICS Consensus Cognitive Battery; PANSS, Positive and Negative Syndrome Scale; CPZ, chlorpromazine equivalents.

** p < 0.01;

*** p < 0.001.

**Figure 1:**
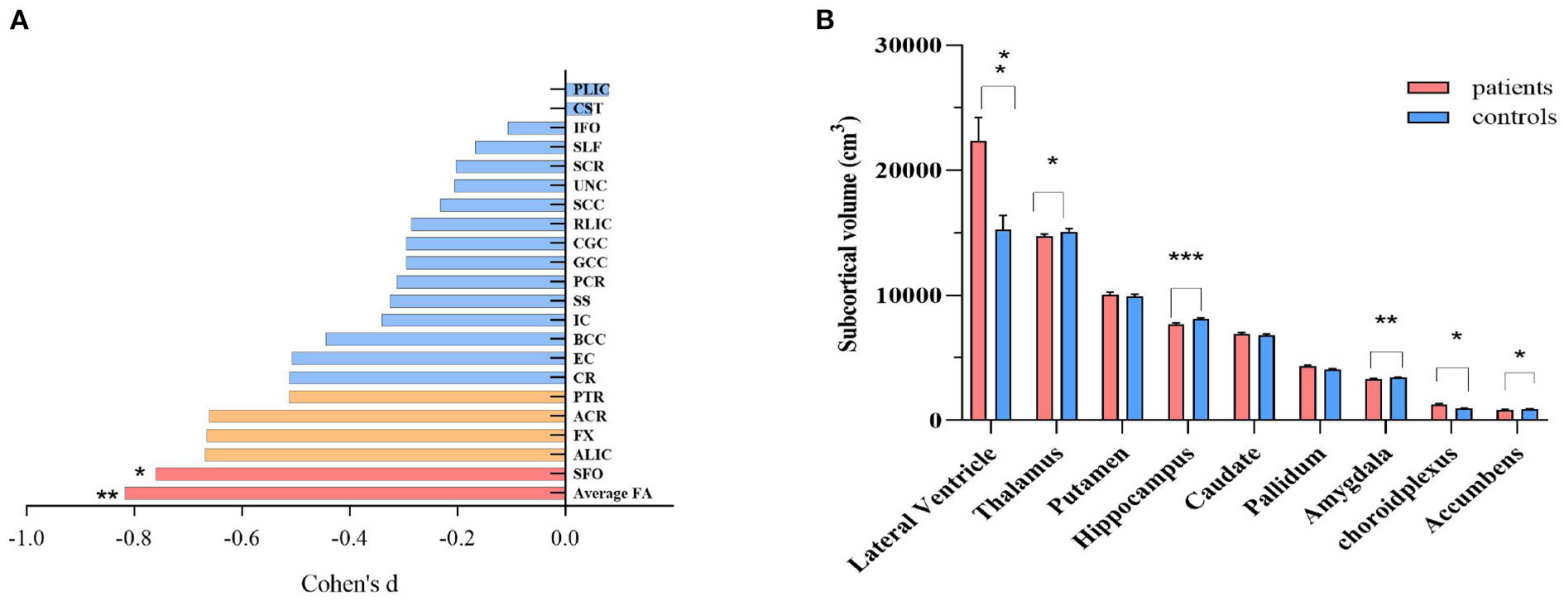
Comparison of regional white matter FA **(A)**, and subcortical gray matter volume **(B)** between schizophrenia patients and healthy controls. ANCOVA was conducted in our study, with age, sex and intracranial volume as covariates. For white matter FA, nominal correlations after multiple comparison (*p* >0.05/21 = 0.002) were drawn in orange. ACR, anterior corona radiata; ALIC, anterior limb of internal capsule; BCC, body of corpus callosum; CGC, cingulum; CR, corona radiata; CST, cortico-spinal tract; EC, external capsule; FX, fornix; GCC, genu of corpus callosum; IC, internal capsule; IFO, inferior frontal occipital fasciculus; PCR, posterior corona radiata; PLIC, posterior limb of internal capsule; PTR, posterior thalamic radiation; RLIC, retrolenticular limb of the internal capsule; SCC, splenium of corpus callosum; SCR, superior corona radiata; SFO, superior fronto-occipital fasciculus; SLF, superior longitudinal fasciculus; UNC, uncinate fasciculus; SS, sagittal striatum; FA, fractional anisotropy. *p < 0.05; ** *p* < 0.01; *** *p* < 0.001, after Bonferroni correction.

We observed significant reductions in average FA for the whole brain, followed by the superior fronto-occipital fasciculus (*p* < 0.05/21 = 0.002) and anterior limb of the internal capsule, fornix, anterior corona radiata, as well as posterior thalamic radiation (all nominal *p's* < 0.05) in SCS (Figure 1A). For subcortical gray matter volume, the patient group showed enlargement of the choroid plexus and lateral ventricles, but reductions of volumes in the thalamus, hippocampus, accumbens, and amygdala subcortical regions, as compared with healthy controls (all *p's* < 0.05/9 = 0.006, Figure 1B). Patients with SCS also had lower average thickness in the left and right hemisphere or average whole-brain cortical thickness as compared to healthy controls (all *p's* < 0.05, Supplementary Figure 1).

### Comparison of the TLR4/NF-κB/IL-1β signaling activit*y* between controls and patients

**Figure 2:**
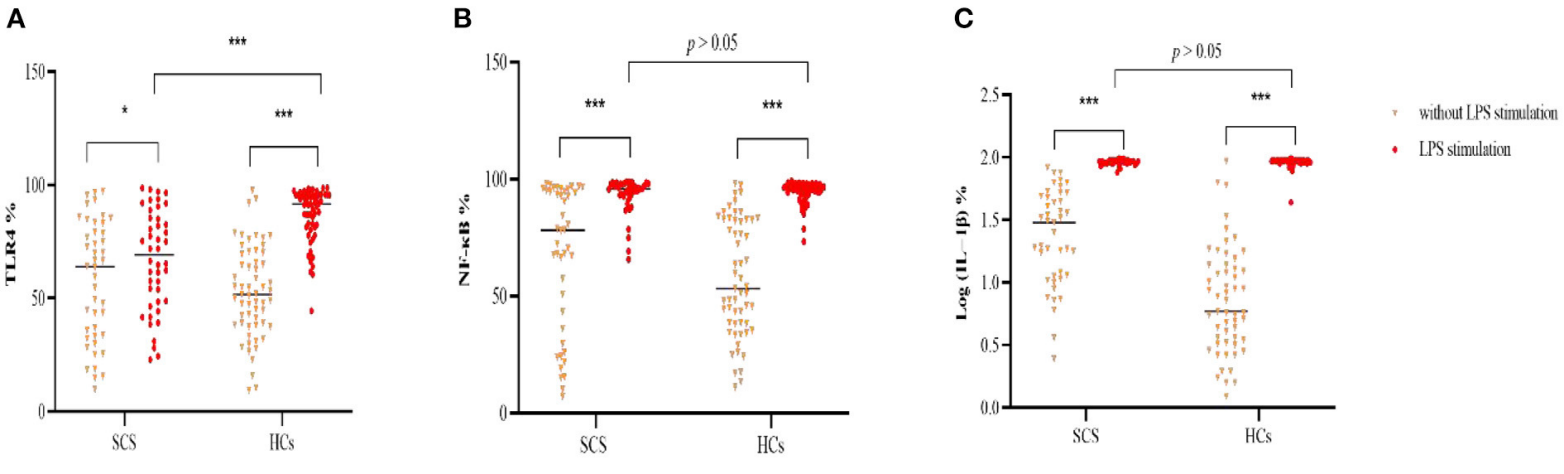
Comparison of percentage of monocytic TLR4 **(A)**, NF-κB **(B)** and log (IL-1β) **(C)** following LPS stimulation between schizophrenia patients and healthy controls. TLR4, Toll-like receptor 4; NF-κB, nuclear factor-kappa B; IL-1β, interleukin-1β; LPS, lipopolysaccharide; %. The percentage of monocytes. *p < 0.05; *** *p* < 0.001.

In an unstimulated state, monocytic IL-1β expression were higher in patients than healthy participants (*p* < 0.05), and there were non-significant but higher trends for the levels of NF-κB and TLR4 in patients with SCS (*p* > 0.05) (Table 2). After LPS stimulation, all of the inflammatory markers were elevated in both groups, but the patient group had a weaker monocytic TLR4 response to LPS stimulation, compared to controls (Figure 2, Supplementary Table 1). And we did not find a statistically significant correlation between CPZ equivalents and TLR4 levels in the patient group (*p* > 0.05).

**Table 2:** The comparison of TLR4 signaling pathway activity in schizophrenia patients and controls.

**%**	**SCS (*n* = 44)**	**HCs (*n* = 59)**	***t***	***p***
TLR4	57.76 ± 26.48	52.98 ± 20.26	−1.08	0.284
NF-κB	67.74 ± 30.65	57.62 ± 24.46	−1.81	0.073
IL-1β	30.03 (11.17–48.72)	5.87 (3.25–13.60)	NA	NA
Log(IL-1β)	1.39 ± 0.36	0.93 ± 0.37	6.22	1.30 × 10^−8^^***^

%, The percentage of monocytes; SCS, Stable Chronic Schizophrenia; HCs, Healthy Controls; NA, Not Applicable.

*** p < 0.001.

### Correlation of TLR4 expression with white matter FA in patients and controls

**Figure 3:**
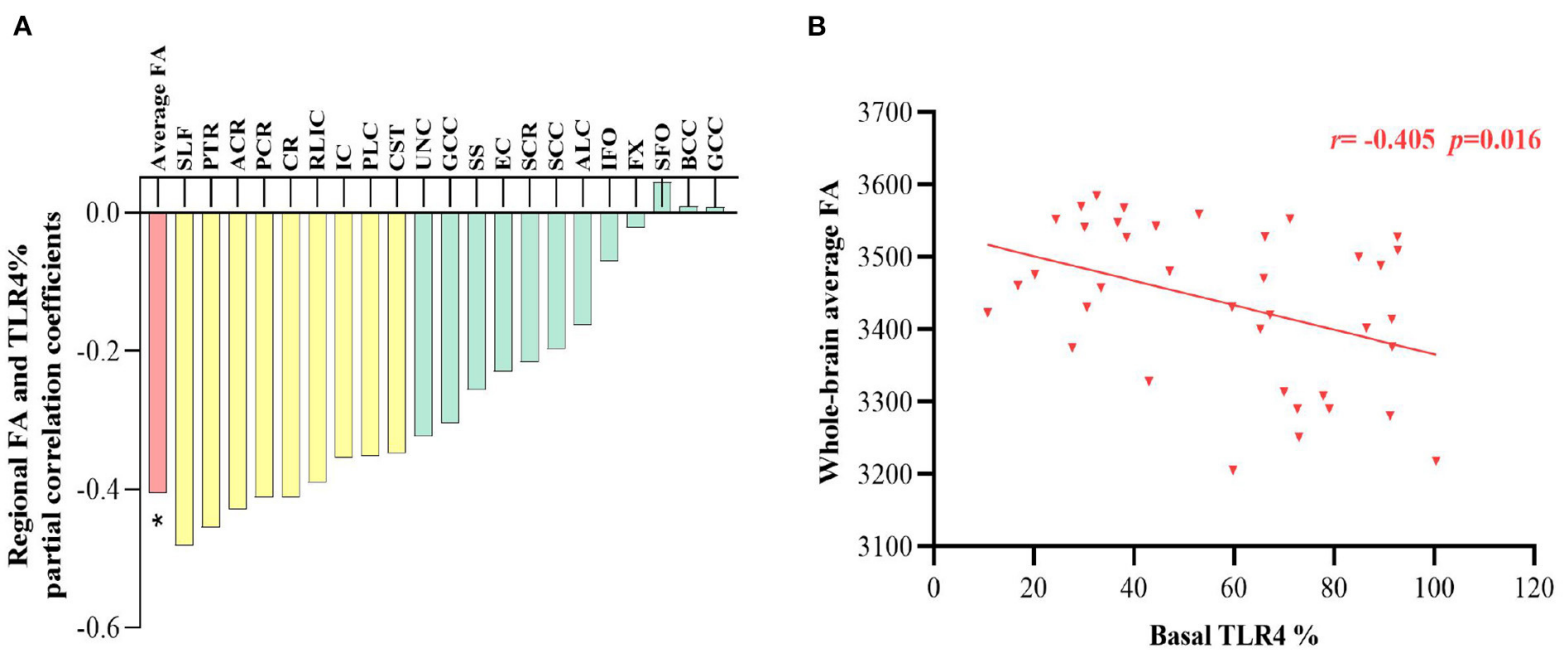
**(A)** Partial correlation coefficients between TLR4% and white matter FA in patients after adjusting for age, sex and intracranial volume, and nominal correlations after multiple comparison (*p* >0.05/21 = 0.002) were drawn in yellow. **(B)** The correlation between TLR4 % and whole-brain average FA is plotted. TLR4, Toll-like receptor 4; FA, fractional anisotropy. %, The percentage of monocytes *p < 0.05.

Partial correlations revealed that basal TLR4 levels significantly negatively correlated with whole-brain average FA (*r* = −0.405, *p* = 0.016, Figure 3) in patients with schizophrenia. Nine specific regions, including the retrolenticular limb of the internal capsule, the anterior corona radiata, corona radiata, cortico-spinal tract, posterior limb of internal capsule, internal capsule, posterior corona radiata, posterior thalamic radiation, and superior longitudinal fasciculus, were nominally inversely correlated with the TLR4 measures (*p* >0.05/21 = 0.002, Figure 3A). All correlations between TLR4 levels and whole-brain average FA or 21 regional FA were not significant in controls (all *p's* >0.05).

### Correlation of TLR4 expression with cortical thickness and subcortical volumes in patients and controls

We also explored subcortical regional volumes as well as cortical thickness and their correlations with TLR4 levels. The results showed that none of cortical structures was significantly correlated with TLR4 levels in either group (all *p's* > 0.05). Neither group showed TLR4 levels that were correlated with 68 regional or whole-brain average cortical thickness (all *p's* > 0.05).

### Correlation of TLR4 expression with cognitive function in patients and controls

**Figure 4:**
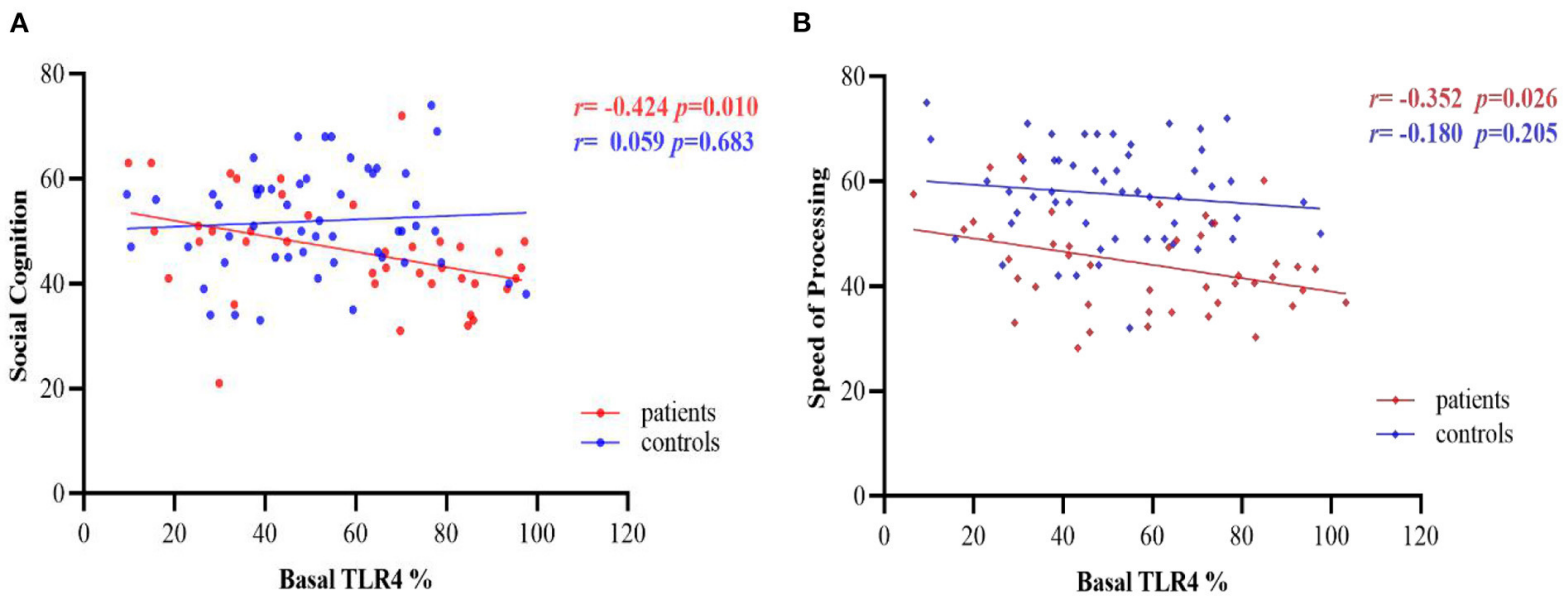
Partial correlations between basal TLR4 % and cognitive performance in patients and controls group, after adjusting for age, sex, education level and CPZ equivalents. **(A)** Correlations between basal TLR4 levels and social cognition; **(B)** Correlations between basal TLR4 levels and speed of processing. TLR4, Toll-like receptor 4. %, The percentage of monocytes.

There were negative correlations between basal TLR4 levels and social cognition and speed of processing of MCCB in the patient group (*r* = −0.424*, p* = 0.010*; r* = −0.352*, p* = 0.026, respectively, Figure 4), with age, sex, education level and CPZ equivalents as covariates, but the results did not pass Bonferroni correction (*p* >0.05/7 = 0.007). No significant correlation was found between TLR4 levels and any cognitive domain functions in the controls (all *p's* >0.05, Figure 4).

### Correlation of TLR4 expression with psychopathology in patients

We also performed partial correlation analysis between TLR4 signaling pathway and PANSS score, with age, sex, and CPZ equivalents as covariates. However, we did not find any statistically significant correlation between TLR4 levels and PANSS-positive score (*p* = 0.595), PANSS-negative score (*p* = 0.060), PANSS-general score (*p* = 0.153) or PANSS total score (*p* = 0.090).

### Correlation of white matter FA with cognition function in patients

We further examined the relationship between cognitive function and whole-brain average as well as individual regional FA to explore whether cognitive decline caused by TLR4/NF-κB signaling activation is due to specific white matter defects or if other brain structures are also involved. After controlling for age, gender, and education level, the whole-brain average FA was found to be significantly and positively correlated with the processing speed of MCCB alone in the patient group (*r* = 0.388, *p* = 0.021, Supplementary Figure 2). However, the correlation did not pass Bonferroni correction at *p* > 0.05/147 (for 21 regions × 7 domains). The nominal correlation coefficients for the other regions varied from −0.355 to 0.474 (*p* = 0.004–0.05).

### Mediation analysis

According to the results of correlation analysis, we used SPSS's PROCESS macro for mediation effect analysis. In the mediation model, we took TLR4 level as an independent variable; and processing speed of MCCB as a dependent variable and average FA as a mediator, respectively. Age, gender, education level and ICV were controlled as covariates. In this study, the 95% CI for total, indirect and direct effects were (−0.31, −0.05), (−0.05, 0.09), (−0.34, −0.05), respectively, suggesting that there was no mediation effect.

## Discussion

Our major findings showed that: (a) schizophrenia patients showed higher activity of the TLR4/NF-κB/IL-1β signaling pathway but dampened monocytic response to LPS stimulation, as compared to healthy controls; and (b) higher TLR4 levels may contribute to cognitive impairment by possibly affecting the white matter rather than gray matter volume or cortical thickness.

Our findings showed that the TLR4 pathway was activated in stable chronic schizophrenia. Monocytic IL-1β, identified as a downstream inflammatory mediator of the TLR4 pathway, was significantly elevated in SCS than healthy subjects. There was a statistical trend toward increased expression of baseline TLR4 and NF-κB in monocytes. Together, these findings suggest a disturbance of the immune system in schizophrenia. We speculated that higher TLR4/NF-κB/IL-1β signaling may compensate for functional deficits of monocytes and, as a result, less increases in monocytic TLR4 levels upon LPS stimulation in schizophrenia. With regard to monocyte/macrophage function, neopterin, a macrophage activation marker, was demonstrated to be decreased in patients with schizophrenia (31). The increased number of monocytes may compensate for cellular function defects (17, 31). Monocyte-derived microglia were also found at a high level in the brain, as can be measured by positron emission tomography (32). Two mechanisms may perhaps explain the origin of TLR4 over-activation: (1) The “leaky gut” hypothesis of schizophrenia: Gram-negative bacteria is translocated into the blood with the increase of intestinal permeability, thus activating TLR4 signaling pathway (33). (2) maternal immune activation induced by prenatal infection including virus, bacteria and protozoan, leading to nitrosative or oxidative stress response (8), which needs further investigation. Indeed, TLR4-induced low-grade inflammation in innate immunity has been well-established in first-episode (16, 31) and chronic schizophrenia (17) and modulating TLR4 pathway activity may have potential therapeutic implications (8).

The current study also indicated an impaired TLR4 signaling pathway activation after LPS challenge, as manifested in lower TLR4 elevation in schizophrenia, consistent with previous reports (17, 31, 34). Impaired TLR4 signal pathways may result in a decreased capability of the body recognizing and clearing invading pathogens *in vivo*, causing sustained mild infection in schizophrenia (16, 18). Nearly half of schizophrenia patients suffered from iatrogenic infections, suggesting that infection may be related to developing schizophrenia (35). The mechanisms underlying infection and immunoreaction have to be clarified in a larger scale cohort study.

Reductions in global average and regional FA were in line with an earlier, large-scale study from the ENIGMA of schizophrenia patients (36). Notably, monocytic TLR4 expression was significantly correlated with the decreases in white matter FA in schizophrenia, inter-linking white matter deficits with impairment in oligodendrocyte function and axonal myelination (37). In the CNS, glial cells support myelination of axons, nutritional supply to neurons, and defense against foreign substances, collectively to ensure parenchymal white matter integrity (38–40). Circulating TLR4-mediated-proinflammatory cytokines, such as IL-1β, IL-6, may mediate peripheral-CNS interaction through impaired blood-brain barrier and enlarged choroid plexus (41), leading to microglia with stronger responses to new inflammatory mediators and as a result, more severe neuroinflammation (42) and neuronal damage (43). Higher TLR4 levels were also found in circumventricular organs, blood brain barrier, leptomeninges, and plexus choroideus in the CNS (8). The finding that TLR4 signaling activation was associated with neuroinflammation accorded with an animal experiment where knockdown of TLR4 blocked TLR/NF-κB and MAPK signaling pathway along with the downstream inflammatory components in astrocytes (44). Microglial inflammation also drives hypomyelination in the brain, contributing to developmental white matter impairment (38), dysfunctional neuroplasticity and networks connectivity (38–40).

We observed subcortical volume differences between patients and controls, including enlargement of the choroid plexus, lateral ventricle, and reduction of thalamus, hippocampus, accumbens, and amygdala volumes, consistent with a large-scale meta-analysis from the ENIGMA consortium (45). Furthermore, lower global and regional cortical thickness was also observed in the patients, in accord with a previous report (21). On the other hand, although neuroinflammation may lead to altered regional brian volumes (46, 47), we did not find a significant relationship between monocytic TLR4 levels and cortical or subcortical volumes in the patients. A likely explanation is that the TLR4 is predominantly expressed on microglial cells and rarely detected on the surface of neurons (48). Microglial activation was reported to be more prominent in white than gray matter in neuropsychiatric disorders, including schizophrenia, Alzheimer's disease, primary progressive aphasia (23, 49, 50). Saijo et al. reported that microglia was more sensitive to LPS stimulation, inducing massive inflammatory mediators; conversely, neurons were relatively insensitive (51). One should also note that antipsychotic medications have anti-inflammatory properties and might reduce microglia activation (32). Together, we speculated that neuroinflammation preferentially affected the white matters in the mild psychopathology of schizophrenia and might progress into a widespread region such as gray matter volume, causing accelerated deterioration from illness.

We observed cognitive impairment in the patients in all seven domains of the MCCB (16, 17, 52). Sustaining low-grade inflammation might account for cognitive deterioration, as shown in both prospective epidemiologic and cross sectional studies (11, 53). Indeed, partial correlation analysis indicated that elevated basal TLR4 levels were distinctly harmful to cognitive function in SCS, replicating a previous study associating higher cell-surface TLR4 levels with cognitive dysfunction in chronic schizophrenia patients (17). Persistent inflammation may enhance the permeability of blood brain barrier (47), allowing peripheral monocyte-derived macrophages to penetrate the CNS and differentiate into microglia cells (54). Additionally, circulating pro-inflammatory mediators such as TNF-α, IL-6 and IL-1 may also interact with the brain through endocrine, neurological, and humoral pathways, exerting complex effects on cognitive function, emotion, and behavior (55). Inflammation in the medial temporal lobe impaired spatial memory in humans (56). Some studies have demonstrated that antipsychotics can affect TLR4 levels (12, 31, 57) and cognitive function (58–60) in patients with schizophrenia. For example, a study of 266 schizophrenic patients found that deficit of overall cognition were significantly related to high CPZ equivalents (58). The finding that the correlations between TLR4 levels and cognition performance did not survive Bonferroni test may possibly be explained by the effects of antipsychotics in this chronic, stable cohort. But the role of TLR4 signaling in cognitive function between first-episode and chronic schizophrenia is inconsistent, presenting a context-dependent pattern (16, 17). Chen et al. (16) pointed out that TLR4 level was positively correlated with cognitive performance in patients with first-episode schizophrenia. The TLR4 signaling pathway may have a dual effect on cognitive function in schizophrenia, manifesting as both benefiting cognitive function and impairing cognitive performance. The balance between rapid immune response and long-term persistent low-grade inflammation and the mechanisms underlying their association warrants further exploration.

We also attempted to explore the relationship between TLR4 levels and psychopathology in patients with schizophrenia. However, we did not find a statistically significant correlation between TLR4 levels and PANSS score. The classical neurotransmitter hypothesis indicated that the imbalance of dopamine level and dopamine receptor activity in the brain is one of the main reasons causing schizophrenia. Hyperfunction of dopamine neurotransmitters in the striatum leads to positive symptoms, while hypofunction of dopamine in the frontal cortex is involved in negative symptoms (61). Considering that TLR4-mediated neuroinflammation preferentially affects glial cells such as microglia and astrocytes, whereas it is rarely or undetectable in neurons (48). Therefore, we speculated that the TLR4 signaling system rarely affects dopaminergic neurons in chronic stable schizophrenia, which may explain our lack of correlation between TLR4 levels and psychopathological symptoms. Later follow-up observations are required to demonstrate this initial hypothesis.

We examined the correlation between cognitive performance and white matter FA, aiming to confirm the hypothesis that TLR4-mediated inflammation may indirectly affect cognitive function by influencing white matter integrity in schizophrenia. We showed that processing speed was positively correlated with the average FA (i.e., response time in negative correlation with FA), suggesting that higher TLR4 signaling could potentially affect the white matter integrity but not gray matter volume or cortical region to affect cognitive performance. Previous studies of neurodegenerative disorders, including Alzheimer's disease and primary progressive aphasia, showed that microglial activation was more prominent in the white than gray matter (49, 50). White matter microstructural dysconnectivity has been related to cognitive impairment and psychopathology in schizophrenia (37, 62). Thus, a blockade of the TLR4 signaling pathway may represent a novel therapeutic strategy for modulating cognitive performance in schizophrenia.

There were inevitably some limitations in this study. First of all, to further explore the causal relationship among the TLR4 signaling pathway, cognitive performance and whole-brain average FA, we attempted to run a mediation effect analysis. Although no mediating effect was found in this study, it may be related to the small sample size. Our study revealed that there were important correlations between TLR4 signaling pathway and cognitive function, white matter FA, and there was also a correlation between white matter FA and cognitive performance in stable chronic schizophrenia, respectively. TLR4-mediated neuroinflammation preferentially affects glial cells such as microglia oligodendrocytes (48), which mainly constitute white matter microstructure and white matter microstructural dysconnectivity has been related to cognitive impairment in schizophrenia (37). Based on the above findings, we initially speculated that TLR4 levels affect cognitive function by affecting white matter integrity. In future studies, a larger sample size is needed to demonstrate our hypothesis, which is the main limitation of this study. Secondly, TLR4 signaling molecules were quantified for peripheral monocytes and their levels reflect at best an indirect measure of neuroinflammation in the brain. A PET study may be used to evaluate TLR4-medicated neuroinflammation in the CNS. Finally, antipsychotic drug exposure may represent a confounding factor, although we recruited patients with stable drug dosing, and did not find a significant correlation between TLR4 levels or white matter FA and CPZ medication (36). The current findings need to be confirmed in drug naïve first-episode schizophrenia.

In conclusion, the current study showed that TLR4/NF-κB/IL-1β signaling elevates in activity but responds sluggishly to LPS stimulation in schizophrenia. Higher TLR4 levels may contribute to cognitive impairment by affecting white matter microstructure rather than subcortical volumes or cortical thickness in schizophrenia.

## Data availability statement

The original contributions presented in the study are included in the article/Supplementary material, further inquiries can be directed to the corresponding author.

## Ethics statement

The studies involving human participants were reviewed and approved by the Ethics Committee of Beijing Huilongguan Hospital. The patients/participants provided their written informed consent to participate in this study.

## Author contributions

YT, YL, and LT designed the project and obtained the funding for this study. WF, WL, SP, JH, HL, NL, MG, JT, YZ, TX, TY, PZ, and WC were responsible for recruiting patients, performing clinical ratings, neuroimaging, and collecting samples. HL analyzed all the data and wrote the paper. YT, SC, and LT are responsible for the integrity of data and the accuracy of data analysis. BT, ZW, ST, XL, and C-SL were invited in evolving the ideas and editing the manuscript. All authors have contributed to and have approved the final manuscript.

## Funding

This work was supported by the Capital Health Research and Development of Special (2022-1-2131), the Beijing Natural Science Foundation (7212054), the National Natural Science Foundation of China (82171507), the Estonian Research Council-European Union Regional Developmental Fund Mobilitas Plus Program (MOBTT77) and the Estonian Research Council personal research funding team grant project (PRG878).

## Conflict of interest

The authors declare that the research was conducted in the absence of any commercial or financial relationships that could be construed as a potential conflict of interest.

## Publisher's note

All claims expressed in this article are solely those of the authors and do not necessarily represent those of their affiliated organizations, or those of the publisher, the editors and the reviewers. Any product that may be evaluated in this article, or claim that may be made by its manufacturer, is not guaranteed or endorsed by the publisher.
