# Supplementary material for: The relationship between TLR4/NF-κB/IL-1β signaling, cognitive impairment, and white-matter integrity in patients with stable chronic schizophrenia
Source: Front Psychiatry. 2022 Aug 16;13:966657. doi: 10.3389/fpsyt.2022.966657 (PMC9424630; doi:10.3389/fpsyt.2022.966657)
Supplement: Supplementary file 1 [file Data_Sheet_1.docx]

Supplementary table1 .Cytokines expression in CD14+monocytes in patients and controls.

| % | SCS (n=44) | | HCs (n=59) | |
| --- | --- | --- | --- | --- |
|  | US | S | US | S |
| TLR4 | 57.76 ± 26.48 | 66.93± 22.04^*^ | 52.98 ± 20.26 | 87.13 ± 11.77 ^***^ |
| NF-κB | 67.74 ± 30.65 | 93.18 ± 7.60 ^***^ | 57.62 ± 24.46 | 94.22 ± 4.92 ^***^ |
| IL-1β | 31.84 ± 22.44 | 89.99 ± 11.68 ^***^ | 11.86 ± 16.41 | 89.32± 17.22 ^***^ |

Abbreviations: %: The percentage of monocytes; US, Unstimulated, without LPS stimulation; S: Stimulated, LPS stimulation; Abbreviations: TLR4, Toll-like receptor 4; NF-κB, nuclear factor-kappa B; IL-1β, interleukin-1β; ^*^*p* < 0.05; ^**^ *p* < 0.01; ^***^ *p* < 0.001.

Supplementary figure1


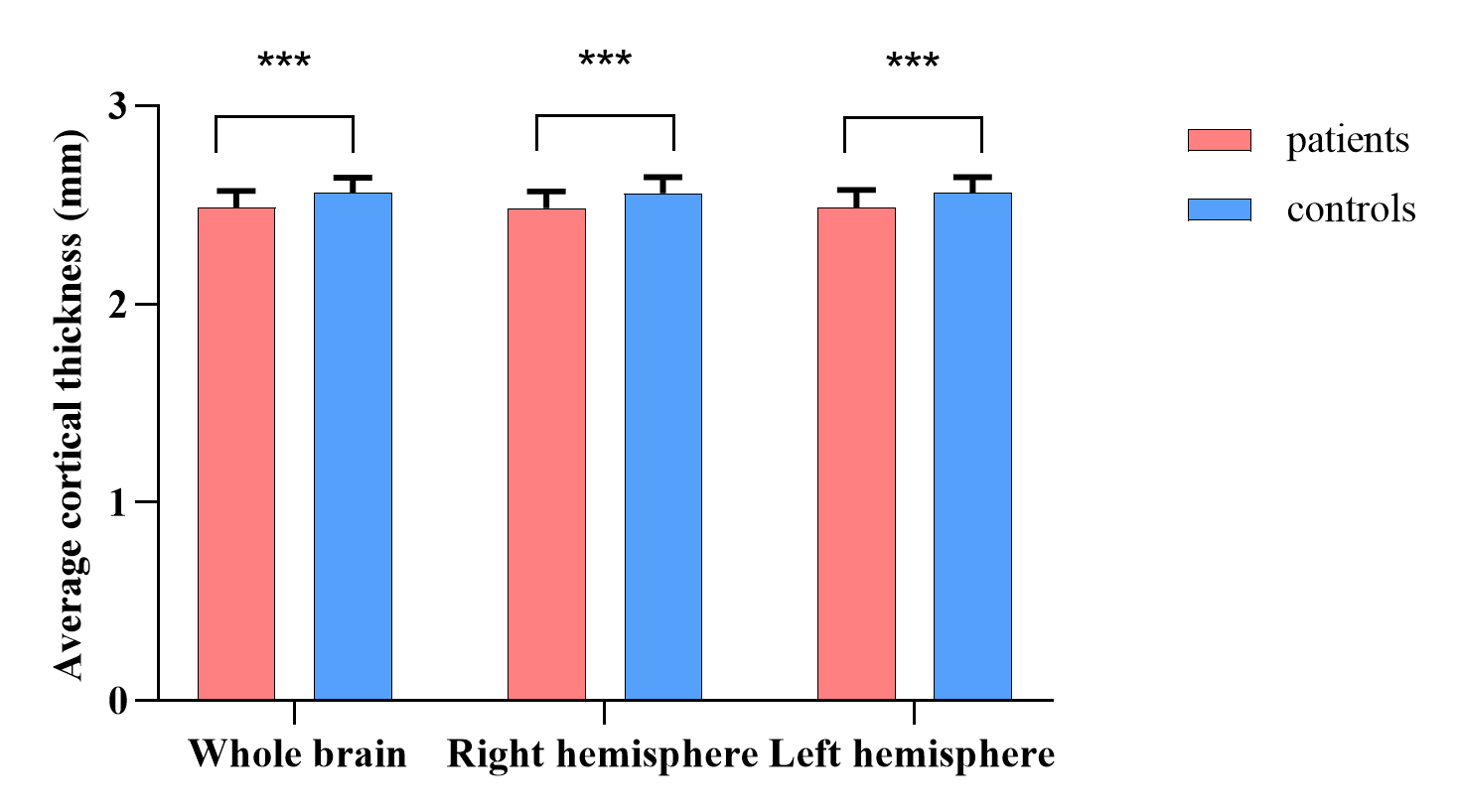
Comparison of cortical thickness between schizophrenia patients and healthy controls. ANCOVA was conducted in our study, with age, sex, and intracranial volume as covariates.^*^*p* < 0.05; ^**^ *p* < 0.01; ^***^ *p* < 0.001, after Bonferroni correction.

Supplementary figure2


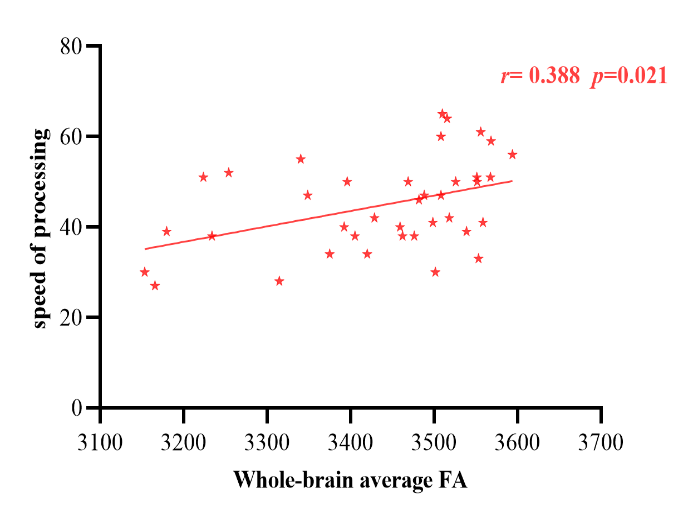


Partial correlations between whole-brain average FA and speed of processing of MCCB in patient group, after adjusting for age, sex, education level, and intracranial volume. Abbreviations: TLR4, Toll-like receptor 4; FA, fractional anisotropy.
