# Supplementary material for: The relationship between TLR4/NF-κB/IL-1β signaling, cognitive impairment, and white-matter integrity in patients with stable chronic schizophrenia
Source: Front Psychiatry. 2022 Aug 16;13:966657. doi: 10.3389/fpsyt.2022.966657 (PMC9424630; doi:10.3389/fpsyt.2022.966657)
Supplement: Supplementary file 2 [file Data_Sheet_2.docx]

Supplementary table 2 Main reagent information

| Reagent | Coding | Company name |
| --- | --- | --- |
| PMG FITC Mouse Anti-Human CD14 | 555397 | BD Biosciences |
| PMG PE Mouse Anti-Human TLR4 | 564215 | BD Biosciences |
| Phosflow™ PE Mouse Anti-NF-kB p65 | 558423 | BD Biosciences |
| Anti-Human IL-1β PE | 12701881 | eBioscience |
| PMG PE Mouse IgG1, κ Isotype Control for TLR4 | 554680 | BD Biosciences |
| PMG PE Mouse IgG2b, κ Isotype Control for NF-κB | 555058 | BD Biosciences |
| PMG PE Mouse IgG1, κ Isotype Control for IL-1β | 559320 | BD Biosciences |
| Lipopolysaccharides (Escherichia coli O55:B5) | L2880-10 | SIGMA-Aldrich |
| Golgistop Protein transport Inhibitor (Containing Monensin) | 554724 | BD Biosciences |
| Phosflow™ Perm/Wash Buffer I | 557885 | BD Biosciences |
| Perm/Wash Buffer | 554723 | BD Biosciences |
| Fixation and Permeabilization | 554722 | BD Biosciences |
| FACS Lysing Solution | 349202 | BD Biosciences |
